# Why Do Some Sex Chromosomes Degenerate More Slowly Than Others? The Odd Case of Ratite Sex Chromosomes

**DOI:** 10.3390/genes11101153

**Published:** 2020-09-30

**Authors:** Homa Papoli Yazdi, Willian T. A. F. Silva, Alexander Suh

**Affiliations:** 1 Department of Biology, Lund University, SE-223 62 Lund, Sweden; 2 Centre for Environmental and Climate Research, Lund University, SE-223 62 Lund, Sweden; willian.silva@evobiolab.com; 3 School of Biological Sciences, University of East Anglia, Norwich Research Park, Norwich NR4 7TU, UK; alexander.suh@ebc.uu.se; 4 Department of Organismal Biology—Systematic Biology, Uppsala University, SE-752 36 Uppsala, Sweden

**Keywords:** sex chromosome, recombination, sexual antagonism, chromatin state

## Abstract

The hallmark of sex chromosome evolution is the progressive suppression of recombination which leads to subsequent degeneration of the non-recombining chromosome. In birds, species belonging to the two major clades, Palaeognathae (including tinamous and flightless ratites) and Neognathae (all remaining birds), show distinctive patterns of sex chromosome degeneration. Birds are female heterogametic, in which females have a Z and a W chromosome. In Neognathae, the highly-degenerated W chromosome seems to have followed the expected trajectory of sex chromosome evolution. In contrast, among Palaeognathae, sex chromosomes of ratite birds are largely recombining. The underlying reason for maintenance of recombination between sex chromosomes in ratites is not clear. Degeneration of the W chromosome might have halted or slowed down due to a multitude of reasons ranging from selective processes, such as a less pronounced effect of sexually antagonistic selection, to neutral processes, such as a slower rate of molecular evolution in ratites. The production of genome assemblies and gene expression data for species of Palaeognathae has made it possible, during recent years, to have a closer look at their sex chromosome evolution. Here, we critically evaluate the understanding of the maintenance of recombination in ratites in light of the current data. We conclude by highlighting certain aspects of sex chromosome evolution in ratites that require further research and can potentially increase power for the inference of the unique history of sex chromosome evolution in this lineage of birds.

## 1. Introduction

Sex chromosomes are the main players in the genetic sex determination of mammals and birds. In eutherian mammals, males are the heterogametic sex, meaning that males carry two morphologically distinct sex chromosomes (male XY), and females are the homogametic sex (XX). In birds, the heterogametic sex is the female (female ZW) and males are the homogametic sex (ZZ). In the mammalian XY system, the *Sry* gene (*Sex-determining region Y*) is located on the Y chromosome and its inheritance to the male embryo determines sex by activating the downstream male regulatory pathway [1]. In birds, the mechanism of sex determination is still under investigation but there is accumulating evidence for the role of *Dmrt1* (*Doublesex- and mab-3-related transcription factor 1*) as the sex-determining gene. *Dmrt1* is located on the Z chromosome and its higher expression in males relative to females drives the formation of male gonads [2]. Sex chromosomes in eutherian mammals and birds have evolved independently, that is, the ancestral autosomes that evolved into sex-chromosomes in birds are not homologous to the ancestral autosomes that evolved into sex-chromosomes in eutherian mammals [3]. However, the common feature in both systems is that the chromosome found only in the heterogametic sex (Y in male heterogamety and W in female heterogamety) is highly degenerated [4], repeat-rich [5,6] and heterochromatic [7]. A key property of Y/W chromosomes is their lack of recombination with the X/Z around the sex-determining region and across the chromosome except for a small segment called the pseudo-autosomal region (PAR) [8,9,10]. 

Several theoretical studies have aimed at explaining the evolution of recombination suppression and its extension beyond the sex-determining region (reviewed in detail in [11]). The most prevalent hypothesis concerns the action of mutations with opposing fitness effects in the two sexes [12,13,14]. According to this theory, the first step involves the establishment of a sex-determining region on a pair of autosomes by restricting recombination between initial sex-determining genes [15]. In the second step, once the sex-determining region is established, cessation of recombination expands further across the chromosome due to the accumulation of sexually antagonistic (SA) mutations. First, SA mutations with a positive fitness effect in one sex and a negative in the other occur in the proximity of the sex-determining region. A recombination-suppressing mutation, commonly assumed an inversion, then tightly links the sex-determining region and SA allele, preventing the damage to the sex in which the SA allele is deleterious [13]. This process is hypothesized to occur in a step-wise manner until recombination is suppressed across most of the chromosome. At each step of recombination suppression, the newly non-recombining region on the Y/W chromosome starts to degenerate [16] due to a multitude of factors, which involves reduction in efficacy of selection because of the linkage of all mutations in the non-recombining segment of the chromosome [17,18,19,20,21]. 

**Figure 1:**
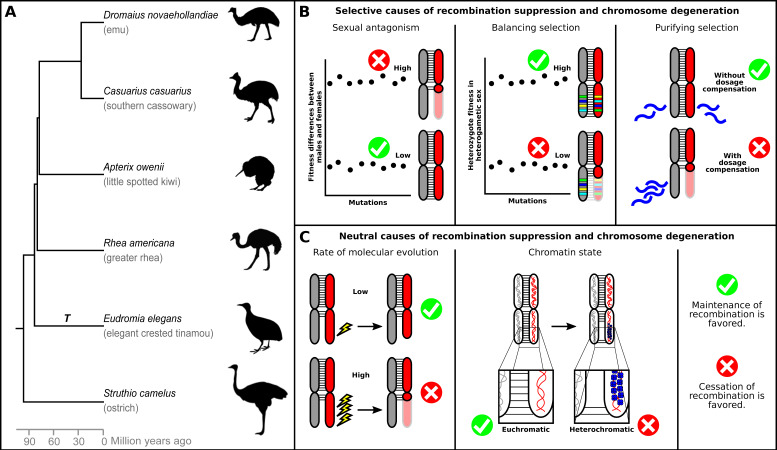
Palaeognathae phylogeny and processes affecting sex chromosome evolution. (**A**) Time tree of the phylogenetic relationships of Palaeognathae drawn after Baker et al. [34] (and consistent with [35]). T: tinamou lineage with heteromorphic sex chromosomes. (**B**) Selective processes that may affect the evolution of recombination rates in sex chromosomes and sex chromosome degeneration: sexually antagonistic selection, balancing selection through heterozygote advantage and purifying selection through lack of dosage compensation. Faded red indicates the original chromosome size. Blue waves in purifying selection represent transcripts. (**C**) Neutral processes that may affect the rate of sex chromosome degeneration: rate of molecular evolution and chromatin state. The slightly smaller W under a low rate of molecular evolution represents a slower degeneration rate. Blue spheres in the heterochromatin represent histones.

In this view of sex chromosome evolution, a progressive suppression of recombination and subsequent loss of genetic material during Y/W degeneration seems to be inevitable. However, the correlation between the age of sex chromosomes and extent of recombination suppression seems to be weak [8]. This can be clearly exemplified in the bird lineage. Birds consist of two major clades, Palaeognathae (tinamous and ratites; Figure 1A) and Neognathae (the remaining >99% of all extant bird species). In Neognathae, sex chromosomes are generally highly differentiated, with the W chromosomes being degenerated, heterochromatic, repeat-rich, and gene-poor [4,22,23,24]. In Palaeognathae, the common karyotypic feature of the flightless ratites (i.e., ostrich, emu, rhea, cassowary and kiwi) is that their Z and W chromosomes are similar in length, undergo recombination across most of their length during meiosis [25,26] and their W is largely euchromatic [27,28,29]. The level of degeneration in the W chromosome in the tinamou lineage, on the other hand, is versatile and the PAR differs significantly in size between different species [30,31] (Table 1). While an exception in birds, across lower vertebrates, homomorphic sex chromosomes exist in many lineages of fish, amphibians and non-avian reptiles. Two hypotheses have been proposed to explain this lack of degeneration. The first concerns sex chromosome turnover which means that new master sex-determining genes are supposed to regularly appear on autosomes, replacing previously established sex chromosomes before they have time to degenerate [32]. The second hypothesis suggests that long-term differentiation is prevented by occasional XY (or ZW) recombination [33]. According to the “fountain-of-youth” model, this homomorphy results from occasional events of sex reversal. In this model, recombination in sex-reversed XY females prevents the long-term degeneration of the Y chromosome. There has not been, to the best of our knowledge, an evidence for sex reversal or sex chromosome turnover in ratites and tinamous.

The striking difference in the state of W degeneration between Neognathae and ratites is particularly puzzling since sex chromosomes in these two lineages share a common ancestor [3]. The sex-determining gene, *Dmrt1*, is located on the Z chromosome in both lineages and is absent not only from the gene-poor W chromosome of Neognathae, but it has also been lost from the W chromosome in ratites [38]. In fact, dating the timing of recombination suppression using the level of sequence divergence between homologous genes in the non-recombining section of the Z and W chromosomes in ostrich (i.e., gametologous genes), has shown the age of the oldest gametologous genes to be before the split of Palaeognathae and Neognathae [36,37,39]. Molecular data shows that the ancestor of all extant birds lived >100 MYA [40] and avian sex chromosomes are thus ancient, making the maintenance of recombination and low degeneration level of ratite sex chromosomes even more striking. It is possible that such difference in degeneration progression of homologous sex chromosomes might be driven by lineage-specific mechanisms that affect the rate of chromosome differentiation [41]. In this article, we look into selective and neutral processes involved in recombination suppression and chromosome degeneration during sex chromosome evolution, using the largely recombining ancient sex chromosomes of ratites as a case study for disentangling these processes.

## 2. Selective Processes

In this section, we discuss selective processes that can influence the evolution of recombination suppression and its subsequent chromosome degeneration in the context of ratite sex chromosomes (Figure 1B). These selective processes include sexually antagonistic, balancing, and purifying selection. When sexual antagonism is not prevalent or when recombination suppression is not the mechanism employed to resolve it, recombination can be maintained between the sex chromosomes. Under a specific set of conditions, balancing selection in the form of over-dominance can maintain recombination. Finally, purifying selection might hinder the degeneration process in the absence of a mechanism to equalize the amount of gene products after gene loss from the non-recombining chromosome. Below, we discuss each process in more detail.

### 2.1. Sexually Antagonistic Selection

The most prominent hypothesis for the evolution of suppressed recombination between the evolving sex chromosomes involves sexual antagonism [13]. Suppression of recombination between sex chromosomes acts as a mechanism to resolve sexual antagonism by prohibiting the transfer of the sexually antagonistic allele to the sex in which the allele is deleterious. According to this theory, the sequential gain of sexually antagonistic mutations leads to a step-wise selection for recombination suppression, driving the degeneration and eventual loss of genetic material from the non-recombining chromosome. It is therefore reasonable to suggest that (i) a lower level of sexual antagonism, (ii) a lower density of functional sequence that can affect fitness upon mutation surrounding the sex-determining region, (iii) a reduced possibility of establishing genetic association between the sexually antagonistic mutations and the sex-determining region, and finally, (iv) an alternative mechanism to resolve sexual antagonism rather than suppression of recombination might have led to the maintenance of recombination in ratites. 

For mutations to have a sexually antagonistic effect, there must be phenotypic variation between sexes that antagonistic selection can act upon. Estimating the prevalence of sexual antagonism within a species is challenging. However, if mating system can be taken as a proxy for the intensity of sexual selection, various mating systems, from monogamy to harem formation with intense male competition, exist among ratites as they do in birds with differentiated sex chromosomes [42,43] (Table 2). Hence, whether levels of sexual antagonism in ratites are particularly different compared to other birds is not clear and requires careful investigation. Furthermore, an important factor to be considered is the antagonistic potential in the PAR, particularly in the proximity of the sex-determining region. The antagonistic potential indicates whether mutations are likely to affect fitness, and more specifically cause SA fitness differences. For a given mutation to be able to have any fitness effect, including sexually antagonistic, the PAR should contain functional regions such as genes or regulatory elements. The map of the Z chromosome in ostrich is available and *Dmrt1* is located at 76.2 Mb near the chromosome end at 80.9 Mb [25]. In chicken, *Dmrt1* is located at 25.9 Mb. Although the gene number and content is conserved between different avian lineages [36], the sequence context surrounding the *Dmrt1* and its relative location to the chromosome centromere might have an influence on the rate of degeneration of the chromosome. 

In the presence of sexual antagonism, SA mutations can only accumulate on the sex chromosome if a non-random association (i.e., linkage disequilibrium (LD)) is established between the sex-determining region and the SA mutation. Without LD, it is not possible for the SA mutation to be associated with the sex it favors. Therefore, one way to prevent the accumulation of sexually antagonistic mutations and the consequent recombination suppression is selection for a higher rate of recombination in the PAR of the heterogametic sex close to the sex-determining region [8]. This can disrupt the establishment of LD between the sex-determining region and the SA alleles and break the cycle of further recombination suppression [8]. The genetic map of ostrich Z chromosome showed that there is potentially a higher recombination rate on the PAR adjacent to the sex-determining region in females compared to males [25]. However, this is similar to the pattern of recombination rate in other birds in which there is a higher rate towards the end of the chromosome and might not be directly related to selection of higher recombination rate for maintenance of the PAR [47]. 

In the case of the establishment of LD between the sex-determining region and the SA mutation, other mechanisms besides recombination suppression might be able to guarantee the restriction of the SA allele in the sex it favors. One such mechanism is sex-biased gene expression, where a male-beneficial mutation present on the Z or W chromosome gets down-regulated in females, and vice-versa in males. One such example of this is in the emu, where it has been suggested that sex chromosomes have become masculinized due to the presence of male-biased expression of Z-linked genes [39]. In a recent study in which the PAR in emu was identified based on the equal coverage of genomic read data between males and females [23], it was shown that Z genes with higher levels of expression in males were in fact hemizygous with a degenerate W chromosome and the higher expression in males simply reflected its higher expression from two Z copies in males. Therefore, sex-biased gene expression, at this moment, cannot be established as a mechanism to resolve sexual antagonism in ratites. 

### 2.2. Balancing Selection

In the previous section, we discussed that under sexually antagonistic selection, the occurrence of a selected allele loosely linked to the sex-determining region will lead to selection for reduced recombination. In this section, we discuss the model by Otto that shows that, under specific conditions, there can be selection for increased recombination between a selected locus and the sex-determining region [48]. This model considers three loci in a male heterogametic system: a sex-determining region, a bi-allelic selected locus called A, and a bi-allelic modifier locus called M that can modify recombination rate between the sex-determining region and the A locus. In a situation where A is in tight linkage with the sex-determining region on the Y chromosome, selection can increase recombination if the heterozygote genotype for the A locus in males has higher fitness. For this to happen, the same allele of the A locus must be favored on the Y chromosome in males and on the X chromosome in females to maintain the polymorphism for the A locus on the X chromosome. Recombination between the Y and the X in males will then transfer the female-advantageous allele locus A from the Y-bearing sperm to the X-bearing sperm. Therefore, fathers will be able to transfer the favorable allele to their daughters, thereby increasing their fitness. However, this increase in fitness in daughters is transient since daughters will transfer the less favorable allele to their sons, reducing their son’s fitness. There will be selection for an increase in recombination if the benefit to daughters is high compared to the reduced fitness in sons. In addition, since the selective advantage to daughters is transient, there must be a loose linkage between the recombination modifier (M) and the A locus to unlink the modifiers from the sex chromosome they affect. In summary, the model suggests that in the case of over-dominance in the heterogametic sex, where the heterozygote genotype has a higher selective advantage compared to the homozygote genotypes, recombination can be maintained by balancing selection. This model can potentially be used for female heterogamety by switching Y to W and X to Z. However, it is worthwhile to mention that the difference between mammalian and avian sex determination system is that the sex-determining gene is located on the Y chromosome in mammals and is therefore inherited solely through the male line, while in birds, the sex-determining gene is located on the Z chromosome and in every generation, it is inherited to both males and females.

It is possible to speculate about the relevance of this hypothesis for the case of recombination maintenance in Palaeognathae. Recombination across most of the W is maintained across all species of ratites and to various degrees in tinamous, which implies that either balancing selection has acted independently in several lineages or all ratite species harbor ancestral balanced polymorphisms. Since the major lineages of Palaeognathae diverged tens of million years ago from one another [34,49], lineage sorting of polymorphisms through genetic drift can be expected to be completed; therefore, it is unlikely for an ancestral balanced polymorphism to have survived such long divergence time. No study has been done on investigating the prevalence of balancing selection in species of Palaeognathae and even so, specifying the type of balancing selection, whether it is, for example, due to over-dominance or negative frequency-dependent selection, is a formidable task. Population genetics study on the sex chromosomes of ratites can provide us with more a detailed view of the population dynamics of loci in the proximity of the sex-determining region. 

### 2.3. Purifying Selection

Lack of recombination between the sex chromosomes in the heterogametic sex reduces the efficacy of natural selection and eventually leads to the degeneration of the non-recombining chromosome. With a degenerated Y/W chromosome, there will be only one copy of the X/Z-chromosomal content in the heterogametic sex. Reducing number of gene copies (i.e., gene dosage) to half can have severe fitness consequences [50,51]. Fitness cost due to dosage imbalance is a consequence of the relationship between gene dosage and the amount of protein product, commonly estimated by the amount of gene expression [52,53]. If genes located on the section of the X/Z chromosome with no Y/W copy are haploinsufficient, which means a single copy of these genes is not enough to produce the normal or wild-type phenotype [54], there is a need for a mechanism to equalize gene products on sex chromosomes between males and females and with respect to autosomes [55]. Three general mechanisms for dosage compensation have been identified: (i) doubling the expression of the hemizygous chromosome in the heterogametic sex; (ii) reducing the expression to half in the homogametic sex; or (iii) the complete inactivation of one sex chromosome in the homogametic sex [56]. 

Dosage compensation does not necessarily occur uniformly for all genes. In birds, dosage compensation has been shown to act in a gene-specific manner [55]. In chicken and flycatcher, levels of dosage compensation are heterogeneous with a more gene-specific pattern instead of a complete shut-down of one Z chromosome in males [57,58]. In ostrich, an RNA-seq study of brain tissue showed a two-fold higher expression level in males compared to females for Z-linked genes that lack a W gametolog [59]. This lack of dosage compensation was suggested to be a constraint for the progression of sex chromosome degeneration. While dosage compensation might be absent in ostrich, it is worthwhile to note that gene expression varies across time and space [60]; therefore, it is possible that the set of genes shown not to be dosage compensated are actually compensated in a particular critical developmental stage. Furthermore, even if dosage compensation is absent in genes located in the hemizygous Z, ZW differentiation can still progress as long as the W gametolog is intact and functionally active. This would explain why most genes on Y/W are gametologs of dosage-sensitive genes, not female/male beneficial genes [61].

## 3. Neutral Processes

In this section, we discuss neutral processes that are relevant for both recombination suppression and subsequent chromosome degeneration (Figure 1C). Degeneration of non-recombining regions on sex chromosomes occurs through the fixation of deleterious mutations, including deletions of coding (genes) or non-coding sequences ranging from small RNAs to transposable elements to pseudogenes. Therefore, a lower occurrence rate of mutations per generation or a lower fixation rate of such mutations might translate to a slower speed of degeneration of newly non-recombining regions. In addition, chromosomal inversions have largely been considered the genetic modifiers of recombination, which can suppress recombination in a step-wise manner, creating evolutionary strata (i.e., non-recombining regions on the chromosomes), with distinct levels of divergence between Z and W chromosome [62]. Here, we highlight the possibility of recombination suppression to occur in a gradual rather than a step-wise manner due to gradual changes in the chromatin state through heterochromatinization [63]. 

### 3.1. Rate of Molecular Evolution

Ratites have long generation times and large bodies (Table 2), which translate to fewer mutations per year and smaller effective population sizes [64]. Indeed, mitochondrial DNA has been shown to evolve at a higher rate in flying birds than in flightless birds and it is negatively correlated with body size [65]. An analysis of 48 avian genomes (including 1 ratite and 1 tinamou) showed that the ostrich has the slowest neutral substitution rate compared to other birds [66] (i.e., approximately half the rate in tinamou, ~2/3 the rate in large-bodied Neognathae and ~1/3 the rate in small-bodied Neognathae). Likewise, analyses of these 48 genomes for transposable element (TE) accumulation rates as well as overall DNA gain/loss rates suggest that the ostrich has by far the least dynamic genome and a larger assembly size due to slower degeneration than other birds since the split of their shared ancestral lineage [67].

The explanation for this is the “accordion model”, where an accumulation of TEs provides substrates for non-allelic homologous recombination (NAHR) (e.g., leading to deletions, inversions, etc.) and is, therefore, followed by an increased rate of deletions countering the genome size increase. Due to this effect, birds with more TE accumulation end up with smaller genomes [67]. In line with these findings, a recent analysis of 12 Palaeognathae genome assemblies suggests that ratites have accumulated fewer young TEs than tinamous, which lost more old TEs [68]. Why ratites would have lower TE accumulation is unclear at this point—we speculate that it could be either because of a lower input of new TE insertions (lower activity of TEs, e.g., resulting from more efficient TE repressors or lower metabolic stress in the absence of powered flight) or a lower fixation rate of new TE insertions (as discussed above considering effective population size and generation time of ratites), or a complex interplay of these two aspects of TE accumulation. Thus, assuming a slower rate of molecular evolution, measured by single nucleotide polymorphisms (SNPs), DNA deletions, and especially TE insertions, this reduced genomic dynamism provides fewer substrates for NAHR, which is also a key process determining the rate of inversions and other large-scale rearrangements [69]. In contrast to other birds with more dynamic genomes, the ratite Z and W chromosomes would, thus, have had a lower input of mutations (especially through TE insertions and inversions) that cause suppression of ZW recombination and degeneration of the W during their evolutionary history. 

### 3.2. Chromatin State of Sex Chromosomes 

**Figure 2:**
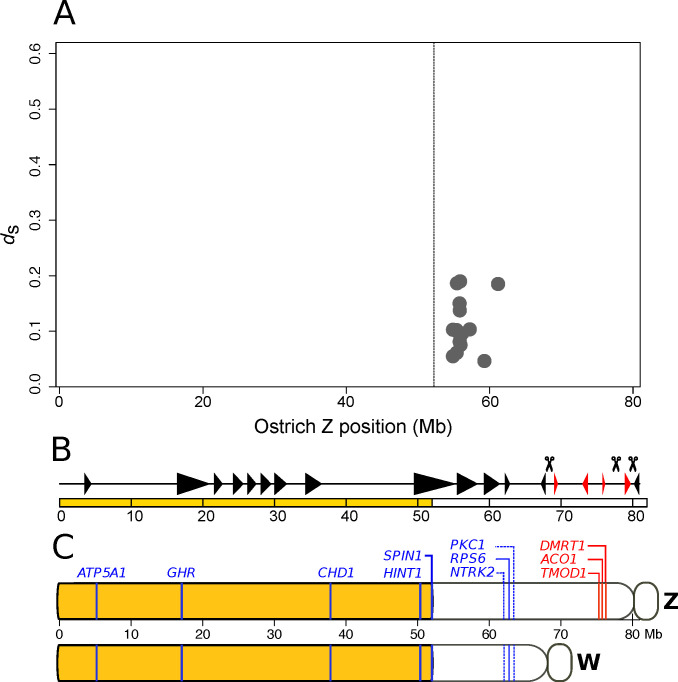
Ostrich sex chromosome evolution. (**A**) Level of synonymous divergence of 16 gametologous genes in ostrich. *d*_s_ estimates for gametologs are obtained from [36]. The vertical line indicates the boundary between the PAR (to the left) and the non-recombining region (to the right). (**B**) Illustration of inverted segments in the ostrich Z chromosome compared to the ancestral state in Neognathae. Red triangles indicate inverted segments and black triangles indicate parallel segments. Scissors represent inversion breakpoints. Figure from Yazdi and Ellegren [25], used under CC BY-NC 4.0 license. (**C**) Schematic representation of the order of genes mapped on Z and W chromosomes using cytogenetic methods [30] and here localized on the ostrich Z chromosome assembly of Yazdi and Ellegren [25]. PAR genes and gametologous genes in blue, hemizygous genes in red, and the yellow segment indicates the PAR.

In addition to discrete and large events, such as inversions leading to evolutionary strata, recombination can be reduced by gradual and small changes [70]. Sixteen of the detected gametologous genes of ostrich with *d*_S_ (synonymous substitutions per synonymous site) between 0.0464 and 0.1898 reside within an about 10 Mb region of the Z chromosome (Figure 2A). In the comparison of the hemizygous region of the ostrich Z chromosome with the ancestral state in lizard, no inversion was detected on the Z chromosome in the region where gametologous genes were identified (Figure 2B) [25]. The comparative cytogenetic map of the ostrich Z and W chromosomes showed that the W gametologs of the three genes located in the non-recombining region, PKCI, RPS6 and NTRK2, are collinear between the Z and the W [30], indicating the absence of inversions (Figure 2C). However, a W chromosome assembly would be needed to have a high enough resolution for excluding the possibility of W-linked inversions in this region. In the absence of large inversions, this would indicate that recombination suppression might have happened through a gradual and gene-by-gene process. 

Gradual suppression of recombination might happen through gradual spread of repressive chromatin states [71]. It is worth noting that repressive histone marks (H3K9me2 and H3K9me3) indicating heterochromatin are not only found in centromeres but are often also deposited in a sequence-specific manner to silence newly inserted TEs [72,73]. This effect can even spread up to 20 kb away from an individual TE insertion in *Drosophila* [74]. Assuming similar heterochromatin dynamics in birds, an increased accumulation of TEs on the W near the PAR boundary (due to low recombination and effective population size), might lead to a positive feedback loop for gradual spread of recombination suppression (see Figure 1 in Kent, et al. [71]). Conversely, assuming that ratites have lower TE accumulation rates (as discussed above considering their slow molecular evolution rate), this feedback loop between TE accumulation, heterochromatin spread and recombination suppression might simply be slower in ratites than other birds. Note, however, that the spread of repressive histone marks, regardless of the cause, may additionally be subject to selective processes acting against changes in gene expression levels. 

Taken together, the slow molecular evolution of ratites and the low degree of heterochromatinization of their W chromosomes might simply reflect that fewer recombination-reducing mutations occurred or became fixed than in other birds. Whether recombination suppression mainly spreads via discrete steps (i.e., through inversions) or gradual changes (i.e., through heterochromatinized TEs) remains to be determined because reference-quality W chromosome assemblies are scarce in birds [24].

## 4. Conclusions

Sex chromosome evolution has been a topic of intense research for several decades. The most common hypothesis to explain recombination suppression (i.e., sexual antagonism) has not provided a conclusive explanation for the observed patterns of recombination suppression and chromosomal degeneration in sex chromosomes [11]. This is not surprising. Sex chromosome evolution, as with most processes in biological systems, is likely not a problem with a single causal variable. A multitude of factors influence recombination suppression and the rate at which it occurs. Moreover, as sex chromosomes become older, it becomes increasingly difficult to disentangle causes and consequences of recombination suppression (i.e., mutations that occur prior to recombination suppression vs. during the degeneration process). It is, therefore, expected that the only way forward is to study such a system from all possible angles. This may not provide definite answers but will allow making inferences with higher confidence about the extent the different processes contributed to sex chromosome evolution. 

To assess the relevance of the sexual antagonism hypothesis in ratite sex chromosome evolution, we must first aim at quantifying levels of sexual conflict in these birds. This of course is another difficult task to achieve. One approach is to compare the mating system and levels of sexual dimorphism in ratites with tinamous and other groups of Neognathae that show various extents of sex chromosome degeneration. Estimates of genetic diversity across the sex chromosomes can also inform us about the presence of sexually antagonistic selection. Theoretical work has shown that levels of genetic diversity are expected to be higher in the PAR region compared to autosomal loci, because the PAR is in linkage to the sex-determining region in the heterogametic sex (female ZW) [75]. The comparison of patterns of genetic diversity in the long PARs of ratites with the values in autosomes will help evaluate if there is any genomic signature of sexual antagonism [76,77]. Furthermore, a valuable resource will be to obtain estimates of recombination rate across the genomes of ratites. It is important to know whether the overall patterns of recombination in ratites resemble that of Neognathae or if their recombination machinery functions in different ways compared to known systems in Neognathae. Finally, gene content around the sex-determining region should be taken into account in understanding the effect of sexually antagonistic mutations. An outstanding question is: Which genes have the potential to create fitness differences between sexes when they mutate? 

It remains unclear how much of recombination suppression occurred in discrete vs. gradual steps. To this end, obtaining high-quality assemblies of Z and W chromosomes of ratites would be instrumental for detecting inversions. Studying the chromatin state of sex chromosomes, particularly the region close to the sex-determining region, using chromatin immunoprecipitation (ChIP) with massively parallel sequencing, will likely provide a better understanding of the impact of repressive histone states of DNA sequences on sex chromosome evolution. Taking all neutral and selective processes into account will reveal whether there is some lineage-specific mechanism that slowed down the rate of sex chromosome recombination suppression and W degeneration in ratites.

## Figures and Tables

**Table 1:** Chromosome number, centromere position, chromatin state, pseudo-autosomal region (PAR) length, and summary statistics of gametologs in Palaeognathae sex chromosomes.

Species	Chromosome Number (2n)	Centromere Position ZW	W Chromatin State	PAR Length	Gametolog *d*_S_ Range	Gametolog *d*_N_*d*_S_ Range
**Ostrich**	80 ^1^	Acrocentric ^4^	Euchromatic ^5^	52.5 Mb ^8^	0.036–2.616 ^6,7^	0.001–0.858 ^6,7^
**Emu**	80 ^2^	Acrocentric ^2^	Euchromatic ^2^	59.3 Mb ^8^	0.128–1.435 ^6^	0.015–0.917 ^6^
**Cassowary**	-	-	-	59.3 Mb ^8^	0.016–0.019 ^8^	0.218–0.273 ^8^
**Little spotted kiwi**	-	-	-	53.6 Mb ^8^	0.002–0.002 ^8^	0.191–0.268 ^8^
**Greater rhea**	82 ^2^	Acrocentric ^2^	Euchromatic ^2^	52.55 Mb ^8^	0.007–0.008 ^8^	0.175–0.225 ^8^
**Elegant crested tinamou**	80 ^3^	Acrocentric ^3^	Heterochromatic with terminal euchromatic segment on its long arm ^3^	32.2 Mb ^8^	0.017–0.706 ^6^	0.001–2.064 ^6^
**Tataupa tinamou**	80 ^3^	Acrocentric ^3^	Completely heterochromatic ^3^	-	-	-

^1^ Takagi et al. 1972 [27]; ^2^ Ansari et al. 1988 [28]; ^3^ Pigozzi 2011 [31]; ^4^ Tsuda, et al. 2007 [30]; ^5^ Nanda, et al. 2008 [29]; ^6^ Zhou, et al. 2014 [36]; ^7^ Yazdi and Ellegren 2014 [37]; ^8^ Xu et al. 2019 [23].

**Table 2:** Life history traits of Palaeognathae lineages.

Species	Mating System	Generation Time (Year)	Sexual Maturity (Year)	Body Mass (kg)
**Ostrich**	Polygynandrous *	16.8 ^†^	3–4 *	100–156 (male) * 90–110 (female) *
**Emu**	Females successive polyandry *	10.5 ^†^	2–3 *	30–55 *
**Cassowary**	Polyandrous *	12.5 ^†^	3 ^‡^	29–34 (male) * 58 (female) *
**Little spotted kiwi**	Monogamous *	9 ^†^	2–3 *	0.88–1.356 (male) * 1–1.950 (female) *
**Greater rhea**	Males simultaneously polygynous, females serially polyandrous *	10.5 ^†^	2–3 *	20–27 *
**White-throated tinamou**	-	6.8 ^†^	-	0.623–0.652 (male) * 0.680–0.8 (female) *

* del Hoyo, J., Elliott, A., Sargatal, J., Christie, D.A. and Kirwan, G. (eds.) (2019). [44] (retrieved from http://www.hbw.com/ on 11 December 2019); ^†^ IUCN 2019. The IUCN Red List of Threatened Species. Version 2019-*3*. http://www.iucnredlist.org. Downloaded on 10 December 2019. [45]; ^‡^ Myers, P., R. Espinosa, C. S. Parr, T. Jones, G. S. Hammond, and T. A. Dewey. 2019. The Animal Diversity Web (online). Accessed at https://animaldiversity.org [46].

## References

[1] Sinclair A.H., Berta P., Palmer M.S., Hawkins J.R., Griffiths B.L., Smith M.J., Foster J.W., Frischauf A.M., Lovell-Badge R., Goodfellow P.N. (1990). A gene from the human sex-determining region encodes a protein with homology to a conserved DNA-binding motif. Nature.

[2] Smith C.A., Roeszler K.N., Ohnesorg T., Cummins D.M., Farlie P.G., Doran T.J., Sinclair A.H. (2009). The avian Z-linked gene DMRT1 is required for male sex determination in the chicken. Nature.

[3] Fridolfsson A.K., Cheng H., Copeland N.G., Jenkins N.A., Liu H.C., Raudsepp T., Woodage T., Chowdhary B., Halverson J., Ellegren H. (1998). Evolution of the avian sex chromosomes from an ancestral pair of autosomes. Proc. Natl. Acad. Sci. USA.

[4] Bellott D.W., Skaletsky H., Cho T.J., Brown L., Locke D., Chen N., Galkina S., Pyntikova T., Koutseva N., Graves T. (2017). Avian W and mammalian Y chromosomes convergently retained dosage-sensitive regulators. Nat. Genet..

[5] Itoh Y., Mizuno S. (2002). Molecular and cytological characterization of SspI-family repetitive sequence on the chicken W chromosome. Chromosome Res..

[6] Saitoh Y., Saitoh H., Ohtomo K., Mizuno S. (1991). Occupancy of the majority of DNA in the chicken W chromosome by bent-repetitive sequences. Chromosoma.

[7] Stefos K., Arrighi F.E. (1971). Heterochromatic nature of W chromosome in birds. Exp. Cell Res..

[8] Otto S.P., Pannell J.R., Peichel C.L., Ashman T.L., Charlesworth D., Chippindale A.K., Delph L.F., Guerrero R.F., Scarpino S.V., McAllister B.F. (2011). About PAR: The distinct evolutionary dynamics of the pseudoautosomal region. Trends Genet..

[9] Smeds L., Kawakami T., Burri R., Bolivar P., Husby A., Qvarnstrom A., Uebbing S., Ellegren H. (2014). Genomic identification and characterization of the pseudoautosomal region in highly differentiated avian sex chromosomes. Nat. Commun..

[10] Hinch A.G., Altemose N., Noor N., Donnelly P., Myers S.R. (2014). Recombination in the human Pseudoautosomal region PAR1. PLoS Genet..

[11] Ponnikas S., Sigeman H., Abbott J.K., Hansson B. (2018). Why Do Sex Chromosomes Stop Recombining?. Trends Genet..

[12] Lenormand T. (2003). The evolution of sex dimorphism in recombination. Genetics.

[13] Rice W.R. (1987). The Accumulation of Sexually Antagonistic Genes as a Selective Agent Promoting the Evolution of Reduced Recombination between Primitive Sex-Chromosomes. Evolution.

[14] Nei M. (1969). Linkage modifications and sex difference in recombination. Genetics.

[15] Charlesworth B., Charlesworth D. (1978). A Model for the Evolution of Dioecy and Gynodioecy. Am. Nat..

[16] Charlesworth B., Charlesworth D. (2000). The degeneration of Y chromosomes. Philos. Trans. R. Soc. Lond. B Biol. Sci..

[17] Muller H.J. (1964). The Relation of Recombination to Mutational Advance. Mutat. Res..

[18] Felsenstein J. (1974). The evolutionary advantage of recombination. Genetics.

[19] Charlesworth B., Morgan M.T., Charlesworth D. (1993). The effect of deleterious mutations on neutral molecular variation. Genetics.

[20] Maynard-Smith J., Haigh J. (1974). Hitch-Hiking Effect of a Favorable Gene. Genet. Res..

[21] Hill W.G., Robertson A. (1966). Effect of Linkage on Limits to Artificial Selection. Genet. Res..

[22] Smeds L., Warmuth V., Bolivar P., Uebbing S., Burri R., Suh A., Nater A., Bures S., Garamszegi L.Z., Hogner S. (2015). Evolutionary analysis of the female-specific avian W chromosome. Nat. Commun..

[23] Xu L., Wa Sin S.Y., Grayson P., Edwards S.V., Sackton T.B. (2019). Evolutionary Dynamics of Sex Chromosomes of Paleognathous Birds. Genome Biol. Evol..

[24] Peona V., Palacios-Gimenez O.M., Blommaert J., Liu J., Haryoko T., Jønsson K.A., Irestedt M., Zhou Q., Jern P., Suh A. (2020). The avian W chromosome is a refugium for endogenous retroviruses with likely effects on female-biased mutational load and genetic incompatibilities. BioRxiv.

[25] Yazdi H.P., Ellegren H. (2018). A Genetic Map of Ostrich Z Chromosome and the Role of Inversions in Avian Sex Chromosome Evolution. Genome Biol. Evol..

[26] del Priore L., Pigozzi M.I. (2017). Broad-scale recombination pattern in the primitive bird Rhea americana (Ratites, Palaeognathae). PLoS ONE.

[27] Takagi N., Ito M., Sasaki M. (1972). Chromosome studies in four species of Ratitae (Aves). Chromosoma.

[28] Ansari H.A., Takagi N., Sasaki M. (1988). Morphological-Differentiation of Sex-Chromosomes in 3 Species of Ratite Birds. Cytogenet. Cell Genet..

[29] Nanda I., Schlegelmilch K., Haaf T., Schartl M., Schmid M. (2008). Synteny conservation of the Z chromosome in 14 avian species (11 families) supports a role for Z dosage in avian sex determination. Cytogenet. Genome Res..

[30] Tsuda Y., Nishida-Umehara C., Ishijima J., Yamada K., Matsuda Y. (2007). Comparison of the Z and W sex chromosomal architectures in elegant crested tinamou (*Eudromia elegans*) and ostrich (*Struthio camelus*) and the process of sex chromosome differentiation in palaeognathous birds. Chromosoma.

[31] Pigozzi M.I. (2011). Diverse stages of sex-chromosome differentiation in tinamid birds: Evidence from crossover analysis in Eudromia elegans and Crypturellus tataupa. Genetica.

[32] Schartl M. (2004). Sex chromosome evolution in non-mammalian vertebrates. Curr. Opin. Genet. Dev..

[33] Perrin N. (2009). Sex reversal: A fountain of youth for sex chromosomes?. Evolution.

[34] Baker A.J., Haddrath O., McPherson J.D., Cloutier A. (2014). Genomic support for a moa-tinamou clade and adaptive morphological convergence in flightless ratites. Mol. Biol. Evol..

[35] Cloutier A., Sackton T.B., Grayson P., Clamp M., Baker A.J., Edwards S.V. (2019). Whole-Genome Analyses Resolve the Phylogeny of Flightless Birds (*Palaeognathae*) in the Presence of an Empirical Anomaly Zone. Syst. Biol..

[36] Zhou Q., Zhang J.L., Bachtrog D., An N., Huang Q.F., Jarvis E.D., Gilbert M.T.P., Zhang G.J. (2014). Complex evolutionary trajectories of sex chromosomes across bird taxa. Science.

[37] Yazdi H.P., Ellegren H. (2014). Old but not (so) degenerated—Slow evolution of largely homomorphic sex chromosomes in ratites. Mol. Biol. Evol..

[38] Shetty S., Kirby P., Zarkower D., Graves J.A. (2002). DMRT1 in a ratite bird: Evidence for a role in sex determination and discovery of a putative regulatory element. Cytogenet. Genome Res..

[39] Vicoso B., Kaiser V.B., Bachtrog D. (2013). Sex-biased gene expression at homomorphic sex chromosomes in emus and its implication for sex chromosome evolution. Proc. Natl. Acad. Sci. USA.

[40] Jarvis E.D., Mirarab S., Aberer A.J., Li B., Houde P., Li C., Ho S.Y., Faircloth B.C., Nabholz B., Howard J.T. (2014). Whole-genome analyses resolve early branches in the tree of life of modern birds. Science.

[41] Schartl M., Schmid M., Nanda I. (2016). Dynamics of vertebrate sex chromosome evolution: From equal size to giants and dwarfs. Chromosoma.

[42] Coddington C.L., Cockburn A. (1995). The Mating System of Free-Living Emus. Aust. J. Zool..

[43] Codenotti T.L., Alvarez F. (2001). Mating behavior of the male Greater Rhea. Wilson Bull..

[44] del Hoyo J., Elliott A., Sargatal J., Christie D.A., Kirwan G.E. (2019). Handbook of the Birds of the World Alive.

[45] IUCN 2019 The IUCN Red List of Threatened Species. Version 2019-3. http://www.iucnredlist.org.

[46] Myers P., Espinosa R., Parr C.S., Jones T., Hammond G.S., Dewey T.A. The Animal Diversity Web. https://animaldiversity.org/.

[47] Kawakami T., Smeds L., Backstrom N., Husby A., Qvarnstrom A., Mugal C.F., Olason P., Ellegren H. (2014). A high-density linkage map enables a second-generation collared flycatcher genome assembly and reveals the patterns of avian recombination rate variation and chromosomal evolution. Mol. Ecol..

[48] Otto S.P. (2014). Selective maintenance of recombination between the sex chromosomes. J. Evol. Biol..

[49] Claramunt S., Cracraft J. (2015). A new time tree reveals Earth history’s imprint on the evolution of modern birds. Sci. Adv..

[50] Torres E.M., Williams B.R., Amon A. (2008). Aneuploidy: Cells losing their balance. Genetics.

[51] Tang Y.C., Amon A. (2013). Gene Copy-Number Alterations: A Cost-Benefit Analysis. Cell.

[52] Khan Z., Ford M.J., Cusanovich D.A., Mitrano A., Pritchard J.K., Gilad Y. (2013). Primate transcript and protein expression levels evolve under compensatory selection pressures. Science.

[53] Ishikawa K., Makanae K., Iwasaki S., Ingolia N.T., Moriya H. (2017). Post-Translational Dosage Compensation Buffers Genetic Perturbations to Stoichiometry of Protein Complexes. PLoS Genet..

[54] Dang V.T., Kassahn K.S., Marcos A.E., Ragan M.A. (2008). Identification of human haploinsufficient genes and their genomic proximity to segmental duplications. Eur. J. Hum. Genet..

[55] Graves J.A.M. (2016). Evolution of vertebrate sex chromosomes and dosage compensation. Nat. Rev. Genet..

[56] Brockdorff N., Turner B.M. (2015). Dosage compensation in mammals. Cold Spring Harb. Perspect. Biol..

[57] Mank J.E., Ellegren H. (2009). All dosage compensation is local: Gene-by-gene regulation of sex-biased expression on the chicken Z chromosome. Heredity.

[58] Uebbing S., Kunstner A., Makinen H., Ellegren H. (2013). Transcriptome sequencing reveals the character of incomplete dosage compensation across multiple tissues in flycatchers. Genome Biol. Evol..

[59] Adolfsson S., Ellegren H. (2013). Lack of Dosage Compensation Accompanies the Arrested Stage of Sex Chromosome Evolution in Ostriches. Mol. Biol. Evol..

[60] Stuart R.O., Bush K.T., Nigam S.K. (2001). Changes in global gene expression patterns during development and maturation of the rat kidney. Proc. Natl. Acad. Sci. USA.

[61] Bellott D.W., Page D.C. (2020). Dosage-sensitive functions in embryonic development drove the survival of genes on sex-specific chromosomes in snakes, birds, and mammals. BioRxiv.

[62] Lahn B.T., Page D.C. (1999). Four evolutionary strata on the human X chromosome. Science.

[63] Huang Y., Liu Q., Tang B., Lin L., Liu W., Zhang L., Li N., Hu X. (2008). A preliminary microsatellite genetic map of the ostrich (*Struthio camelus*). Cytogenet. Genome Res..

[64] Figuet E., Nabholz B., Bonneau M., Carrio E.M., Nadachowska-Brzyska K., Ellegren H., Galtier N. (2016). Life History Traits, Protein Evolution, and the Nearly Neutral Theory in Amniotes. Mol. Biol. Evol..

[65] Lartillot N., Poujol R. (2011). A phylogenetic model for investigating correlated evolution of substitution rates and continuous phenotypic characters. Mol. Biol. Evol..

[66] Zhang G.J., Li C., Li Q.Y., Li B., Larkin D.M., Lee C., Storz J.F., Antunes A., Greenwold M.J., Meredith R.W. (2014). Comparative genomics reveals insights into avian genome evolution and adaptation. Science.

[67] Kapusta A., Suh A., Feschotte C. (2017). Dynamics of genome size evolution in birds and mammals. Proc. Natl. Acad. Sci. USA.

[68] Wang Z., Zhang J., Xu X., Witt C., Deng Y., Chen G., Meng G., Feng S., Szekely T., Zhang G. (2019). Phylogeny, transposable element and sex chromosome evolution of the basal lineage of birds. BioRxiv.

[69] Konkel M.K., Batzer M.A. (2010). A mobile threat to genome stability: The impact of non-LTR retrotransposons upon the human genome. Seminars in Cancer Biology.

[70] Darolti I., Wright A.E., Mank J.E. (2020). Guppy Y Chromosome Integrity Maintained by Incomplete Recombination Suppression. Genome Biol. Evol..

[71] Kent T.V., Uzunovic J., Wright S.I. (2017). Coevolution between transposable elements and recombination. Philos. Trans. R. Soc. B.

[72] Choi J.Y., Lee Y.C.G. (2020). Double-edged sword: The evolutionary consequences of the epigenetic silencing of transposable elements. PLoS Genet..

[73] Hollister J.D., Gaut B.S. (2009). Epigenetic silencing of transposable elements: A trade-off between reduced transposition and deleterious effects on neighboring gene expression. Genome Res..

[74] Lee Y.C.G., Karpen G.H. (2017). Pervasive epigenetic effects of Drosophila euchromatic transposable elements impact their evolution. Elife.

[75] Kirkpatrick M. (2010). How and why chromosome inversions evolve. PLoS Biol..

[76] Kirkpatrick M., Guerrero R.F., Scarpino S.V. (2010). Patterns of neutral genetic variation on recombining sex chromosomes. Genetics.

[77] Kirkpatrick M., Guerrero R.F. (2014). Signatures of sex-antagonistic selection on recombining sex chromosomes. Genetics.

